# The Effect of Blood on Apical Microleakage of Epiphany and AH26: An In Vitro Study

**Published:** 2011-05-15

**Authors:** Zohreh Khalilak, Mehdi Vatanpour, Maryam Javidi, Moravrid Mafi, Farzaneh Afkhami, Farzaneh Daneshvar

**Affiliations:** 1 *Department of Endodontics, Dental School, Islamic Azad University of Medical Science, Tehran, Iran*; 2 *Department of Endodontics, Dental Material Research Center/Dental School, Mashad University of Medical Sciences, Mashad, Iran*; 3 *Dentist, Private Practice, Mashad, Iran*; 4 *Department of Endodontics, Dental School, Mashad University of Medical Sciences, Mashad, Iran*

**Keywords:** AH26, Endodontics, Epiphany Sealer, Microleakage, Fluid Filtration

## Abstract

**INTRODUCTION:** Blood contamination of the canal during preparation can be a major problem in endodontics; this may result in apical microleakage. The aim of this* in vitro *study was to evaluate the effect of blood on apical microleakage of a resin-based root canal sealer (AH26) and a polymer-based root canal sealer (Epiphany).

**MATERIALS AND METHODS:** In this experimental study, 50 decoronated central incisors and canine teeth were prepared by RaCe rotary system and randomly divided into 4 groups (n=10). Groups A_1 _and A_2 _were obturated by Epiphany/Resilon and AH26/Gutta-percha, respectively. The obturations were performed with a single cone technique after drying root canals. In B_1 _and B_2_ groups, the test groups, 0.02cc citrated human blood was injected into dried root canals and they were obturated in the same manner. Ten specimens were served as positive and negative controls (n=5).The apical leakage was measured by means of a computerized fluid filtration method after 1 day and 3 weeks. The data was analyzed by One-Sample Kolmogorov-Smirnov, Independent Sample t-test and univariate analysis. Statistical significances were preset at α=0.05.

**RESULTS:** There was no significant difference in apical microleakage of the two sealers after 1 day and 3 weeks in dry and blood environment (P>0.05). Sealer and environment had no interaction (P>0.05).

**CONCLUSION:** Blood contamination has no significant effect on the apical microleakage of Epiphny and AH26.

## INTRODUCTION

**L**eakage of blood from tooth apical foramen is one of the major reasons that results in microleakage after endodontic treatments (1). According to a Toronto study, 88% of treatment failure is due to apical microleakage (2). The initial ideal properties of root canal sealer was introduced by Grossman *et al.*, and tissue fluid resistance is one of them (3,4). A wide variety of root canal sealers are commercially available (5-8). The effect of moisture and blood on setting process of sealer is very important. Inability of gaining good apical seal results in percolation of blood-borne proteins, microbes, their toxins and other stimulating factors, which can result in inflammation and root canal therapy failure. Many studies have proposed that apical microleakage occurs due to the prior existence of blood and moisture in canal during obturation (7,9,10), but recent studies have claimed that remnant moisture does not alter the mean value of microleakage (1,6,8).

Recently a thermoplastic synthetic polymer-based root canal filling material was introduced. The resin core filling material, Resilon (Resilon Research LLC, Madison CT), functions like gutta-percha. Obturation with Resilon cones is accomplished by use of Epiphany primer (Pentron Clinical Technologies, LLC, Wallingford, CT) and Epiphany resin-based sealer (Pentron Clinical Technologies, Wallingford, CT, USA) (8). One study compared the bacterial leakage of gutta-percha and Resilon and found that Resilon had a mean leakage of 7-13%, whereas gutta-percha leakage values reached 73-93% (11). They attributed the resistance to leakage and increased sealing properties to the "mono-block" created by the affinity of the Resilon core to the Epiphany resin based sealer. Another survey reiterated the mono-block concept in an *in vivo *study with dogs, showing less apical periodontitis in teeth obturated with Resilon (12). Another reason for good results of Epiphany/Resilon in leakage studies maybe due to its dentin bonding agent, which makes the moisture control less critical (13). However, a further study conducted with a transmission electron microscope (TEM) revealed the presence of silver deposits along the sealer hybrid layer interface in Epiphany/Resilon, and between AH-Plus/gutta-percha and concluded that a complete hermetic apical seal cannot be achieved with any of these filling materials (14).

AH26 (DENTSPLY, Detrey, France) is a common epoxy-resin sealer, which is claimed to provide excellent sealing properties (15,16). However, the lack of bonding between gutta-percha and AH26 may allow leakage (16).

Since there was no information available about the differences between sealing ability of Epiphany/Resilon and AH26/Gutta-Percha in a blood contaminated canal, the present study was performed to find the effect of blood contamination on the apical sealing ability of Epiphany and AH26 after 1 and 21 days.

## MATERIALS AND METHODS

Fifty human maxillary central incisors and canine teeth with single straight root canals and fully developed apices were used in this experimental study. Teeth with open apices, cracks, caries and resorptive defects were excluded. The crowns were cut using a water-cooled diamond discs, resulting in approximately 12mm root length. The canal length was visibly established by placing a size #10 K-file (Dentsply/Mailefer, Ballaigues, Switzerland) into each root canal until the tip of the file was visible at the apical foramen. The working length was established at approximately 11mm. The root canal system was instrumented to the working length using crown dawn technique by RaCe rotary system (FKG, Dentaire Co., Dental. Products, Switzerland) up to 06 #40. The root canals were irrigated with 2cc 5.25% NaOCl after each file throughout preparation. The smear layer was removed with 1cc 17% EDTA (Aria Dent, ACT, and Iran) and 1cc 5.25% NaOCl (each was left within the canal for 1 minute). Subsequently, all canals were rinsed with 5cc distilled water.

The specimens were randomly divided into 4 experimental (n=10), and two control (n=5) groups.

In groups A1 and A2 (dry) the canals were dried with five paper points size #25, until the last paper point which was left in the canal for 5 sec remained dry.

In groups B1 and B2 (blood-contaminated) the canals were dried with size #25 paper points, until the last paper point which was left in the canal for 5 sec remained dry. Subsequently, the canals were contaminated with 0.02cc citrated human blood which was injected along the entire working length.

The specimens in groups A1 and B1 were obturated by Epiphany/Resilon (Resilon Research LLC, Madison CT) with one 06 taper #40 Resilon single cone. Epiphany self etching primer was introduced into the root canal with a micropipette provided by the manufacturer and excessive primer was removed with a paper point size #25 according to manufacturer. Sealer was carried to the canal by means of a 06#40 RaCe file. After obturation, coronal part was cured with a light-cure device (Coltene, Whale dent Inc, USA) for 40 seconds. A hot spoon excavator and a heated condenser were used to remove the excessive Resilon up to 1mm below the coronal region of the canal which was then sealed with a light-cure glass ionomer (GC Fuji II, Tokyo, Japan) to ensure coronal seal. Glass ionomer was cured for 40 seconds.

**Figure 1:**
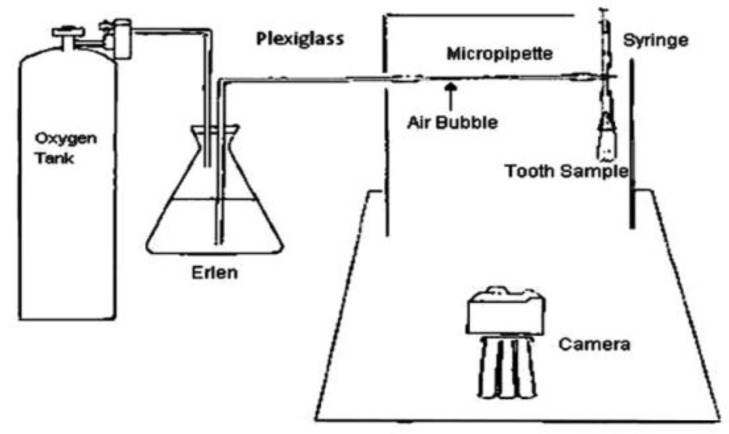
A schematic view of computerized fluid filtration

The specimens from the groups A2 and B2 were obturated with AH26 (DENTSPLY, Detrey, France) and one 06 #40 gutta-percha (DiaDent Group International, Korea) as single cone technique. Sealer was carried to the canal by means of 06 #40 RaCe File. A hot spoon excavator and a heated condenser were used to remove 1mm excess gutta-percha from the orifices; the canal was subsequently sealed with glass ionomer.

Positive controls (n=5) were instrumented but not obturated and the coronal parts were sealed with glass ionomer as the other groups. The negative controls (n=5) were completely covered with two layers of nail varnish (ETUDE Corporation, Korea) and two layers of parafilm stripes (Parafilm "M", Laboratory Film, Chicago, USA).

All of the specimens were placed in a 37^º^c incubator with 100% humidity. After 1 day and 3 weeks the apical microleakage was measured by means of a Computerized Fluid Filtration model designed by Pashley *et al.* and modified by Javidi *et al.* (Figure 1) (17). The data was analyzed using One-Sample Kolmogorov-Smirnov, Independent Sample t-test and Univariate analysis tests with SPSS (Version 16; SPSS Inc, Chicago, IL) software. Statistical significance was preset at α=0.05.

## RESULTS

**Figure 2:**
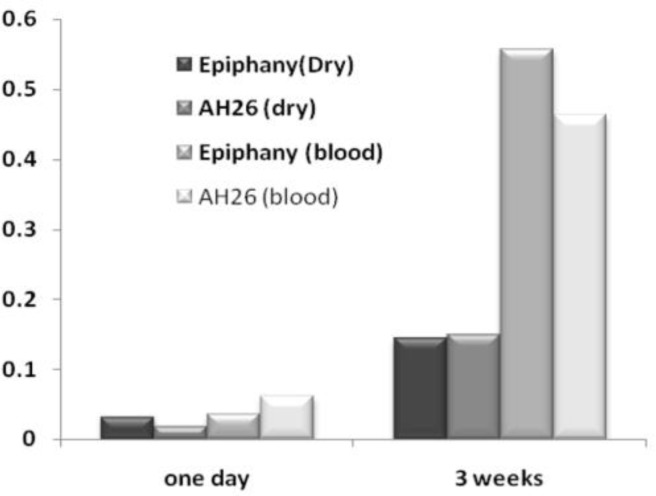
Mean of apical microleakage (µl/min/Cm H2O) for each experimental group after 1 day and 3 weeks

The results of this study are illustrated in Table 1 and Figure 2. Independent Sample t-test showed that the experimental endodontic sealers (AH26 and Epiphany) and dry or blood environments had no statistically significant effect on the mean value of the apical microleakage after one day and three weeks (P>0.05). The mean value of apical microleakage of all groups increased within time from 1 day to 3 weeks. The results of two way ANOVA showed that sealer and environment had no interaction (P=0.259).

## DISCUSSION

Ideally, a root canal sealer should be capable of producing a bond between the core material and the root dentin, to effectively prevent microleakage. Root canal obturation with gutta-percha and sealer is not reliable for providing a long-time seal (18,19). Leakage studies on the sealing properties of endodontic materials are still important and relevant. A wide variety of root canal sealers are commercially available; however there is no consensus on the material which has the most effective seal. In this study, AH26 was used due to the reports of its excellent sealing properties (15,16). The lack of bonding between gutta-percha and AH26 may allow leakage (16). However, in the Epiphany root obturation system, the Epiphany sealer's attachment to the root canal walls and to the Resilon core material seems to be more suitable (20). According to the study by Shipper *et al.* (11) the excellent sealing capability of Epiphany may be attributed to its integrity, which is provided by the adhesion of the Resilon filling material to the Epiphany sealer, and, the Epiphany sealer's adhesion to the dentin walls in the root canal system.

According to our study there was no significant difference in dry and blood environment when either AH26 or Epiphany was used after 1 day and 3 weeks. So if Epiphany/Resilon could have produced a "mono-block", the mean value of apical microleakage should have been lower. Although the concept of creating mechanically homogenous unit with dentin is excellent in theory, accomplishing it by means of a mild self-etch acid (like the Epiphany's adhesive) in the root canal space is complicated to say the least (26-28).

**Table 1:** Apical micro leakage (µl/min/Cm H2O) for each experimental group after 1day and 3weeks (Independent Sample t-test)*.*

**Sealer**	**Environment**	**Time**	**Min**	**Max**	**Mean**	**SD**
Epiphany	Dry	1 Day	0.0023	0.1334	0.03	0.4044
		3 Weeks	0.0163	0.2969	0.14	0.0933
	Blood	1 Day	0.0023	0.1215	0.03	0.04
		3 Weeks	0.0412	2.0201	0.55	0.68
AH26	Dry	1 Day	0.0023	0.0982	0.01	0.28
		3 Weeks	0.0070	1.1456	0.14	0.35
	Blood	1 Day	0.0023	0.2455	0.06	0.07
		3 Weeks	0.0233	1.4355	0.46	0.46

Sometimes, during canal preparation, blood contamination may occur and it can be noticed by blood contamination of spreader or it may be microscopic with no clinical manifestation. In these situations, the best approach is to remove all of the filling materials, stop the bleeding and clean the canal, but this cannot always be achieved. Sometimes due to clinical necessity, it may be logical continue with obturation. So, it is critical to find a sealer with a low apical microleakage in a blood contaminated canal.

Recently, the fluid transport method has been demonstrated to be the method of choice in determining leakage. Leakage can be measured with greater sensitivity with this method than with dye penetration along the root canal. The biggest advantage of the fluid transport model is that it examines leakage without destroying the specimens and is cheaper, easier and more available than protein leakage test (17,20-24).

The removal of smear layer may be considered an essential step in the process of successful root canal treatment (25). For this reason, the smear layer was removed to evaluate the penetration and adaptation of root canal filling material in our study. To avoid anatomical variations and to standardize the leakage measurements in this study, the length of the samples, the canal diameter, and the canal anatomy was kept constant.

Number of reasons have been indicated for the imperfect sealing of Resilon/Epiphany (26) *i.e.* uneven application of the self-etching primer to the root canal, inadequate evaporation of solvent from the primer, uneven application of the sealer to the root canal, inadvertent stripping of the sealer off the canal wall during insertion of cones, and increased C-factor due to extremely limited capacity of a narrow root canal to relieve the stress of polymerization shrinkage created by sealers flow; this may result in debonding of filling material from dentinal tubules and the increase of microleakage (29).

However, according to previous studies (26,30,31) it seems that Epiphany can produce a more superior seal in comparison to AH26 in an ideal environment.

Although Epiphany is a methyl metacrylate based sealer, this study revealed no significant increased apical microleakage when it was used in a blood contaminated environment.

## CONCLUSION

No significant difference was found between apical microleakage of Epiphany and AH26 in a dry or blood contaminated canal after 1day and 3weeks. We have only measured the apical seal; however, the coronal seal in endodontics is as important as apical seal. Further *in vivo *studies are required for more definitive conclusions.
